# Caloric restriction delays age-related muscle atrophy by inhibiting 11β−HSD1 to promote the differentiation of muscle stem cells

**DOI:** 10.3389/fmed.2022.1027055

**Published:** 2023-01-05

**Authors:** Shan Lv, Qianjin Shen, Hengzhen Li, Qun Chen, Wenqing Xie, Yusheng Li, Xiaodong Wang, Guoxian Ding

**Affiliations:** ^1^Department of Geriatric Endocrinology, Jiangsu Province Hospital and Nanjing Medical University First Affiliated Hospital, Nanjing, Jiangsu, China; ^2^Department of Emergency Medicine, Sir Run Run Hospital, Nanjing Medical University, Nanjing, Jiangsu, China; ^3^Department of Orthopedics, Xiangya Hospital, Central South University, Changsha, Hunan, China; ^4^Department of Orthopedics, Jiangsu Province Hospital and Nanjing Medical University First Affiliated Hospital, Nanjing, Jiangsu, China

**Keywords:** caloric restriction (CR), 11β-HSD1, muscle atrophy, muscle stem cells (MuSCs), mitochondrial function

## Abstract

**Introduction:**

Calorie restriction (CR) is an important direction for the delay of sarcopenia in elderly individuals. However, the specific mechanisms of CR against aging are still unclear.

**Methods:**

In this study, we used a CR model of elderly mice with muscle-specific 11β-hydroxysteroid dehydrogenase 1 (11β-HSD1) knockout mice and 11β-HSD1 overexpression mice to confirm that CR can delay muscle aging by inhibiting 11β-HSD1 which can transform inactive GC(cortisone) into active GC(cortisol). The ability of self-proliferation and differentiation into muscle fibers of these mouse muscle stem cells (MuSCs) was observed *in vitro*. Additionally, the mitochondrial function and mitochondrial ATP production capacity of MuSCs were measured by mitochondrial oxygen consumption.

**Results:**

It was found that the 11β-HSD1 expression level was increased in age-related muscle atrophy. Overexpression of 11β-HSD1 led to muscle atrophy in young mice, and 11β-HSD1 knockout rescued age-related muscle atrophy. Moreover, CR in aged mice reduced the local effective concentration of glucocorticoid (GC) through 11β-HSD1, thereby promoting the mitochondrial function and differentiation ability of MuSCs.

**Conclusions:**

Together, our findings highlight promising sarcopenia protection with 40% CR in older ages. Furthermore, we speculated that targeting an 11β-HSD1-dependent metabolic pathway may represent a novel strategy for developing therapeutics against age-related muscle atrophy.

## Highlights

- 11β-HSD1 expression levels were increased in age-related muscle atrophy.
- Overexpression of 11β-HSD1 led to muscle atrophy in young mice, and muscle-specific 11β-HSD1 knockout rescued age-related muscle atrophy.
- CR in aged mice reduced the local effective concentration of glucocorticoid (GC) through 11β-HSD1, thereby promoting the mitochondrial function and differentiation ability of MuSCs.
- Our findings highlight promising sarcopenia protection with 40% CR in older ages.
- We speculated that targeting an 11β-HSD1-dependent metabolic pathway may represent a novel strategy for developing therapeutics against age-related muscle atrophy.

## 1. Introduction

Progressive age-related loss of skeletal muscle mass and function is known as sarcopenia and increases an adult’s individual’s susceptibility to adverse clinical outcomes (1). Currently, there is no effective treatment for sarcopenia, and the treatment is limited to improving nutrition and strengthening exercise. It is well known that muscle stem cells (MuSCs) are mainly responsible for skeletal muscle regeneration (2). With increasing age, the proliferation and differentiation abilities of MuSCs decrease significantly, resulting in impaired muscle regeneration in senescent individuals (3). Thus, improving the function of MuSCs is an effective way to delay sarcopenia.

It is well established that calorie restriction (CR) cannot only reduce fat accumulation and improve insulin resistance but can also maintain organ function and has anti-aging effects (4). Recently, Sharples et al. also found that CR is an effective intervention method to reduce progressive muscle loss in elderly individuals, which is of great significance to prolong life and promote healthy aging (5). Yang et al. found that CR maintained muscle homeostasis and improved muscle protein quality by enhancing autophagy and reducing inflammation, indicating that CR is an important regulator of sarcopenia (6). In addition, studies have found that CR cannot only increase the expression of Pax7, a specific gene of muscle stem cells, but can also increase the number of mitochondria, oxidative respiratory chain enzymes, and aerobic utilization in stem cells (7). Thus, CR is an important direction for the delay of sarcopenia in elderly individuals. However, the specific mechanisms of CR against aging are still unclear.

Numerous studies have shown that long-term stress accelerates aging (8, 9). In fact, glucocorticoid (GC) is a stress hormone, and endogenous GC levels increase by 20–50% with aging (10). The GC activity is mainly regulated by 11β-hydroxylated steroid dehydrogenase1 (11β-HSD1) which can transform inactive GC(cortisone) into active GC(cortisol). So 11β-HSD1 is known as the local amplifier of GC and plays a key role in regulating organ aging (11). Some studies have found that the expression of 11β-HSD1 increases with aging in brain, skin, and muscle tissues, which is closely related to impaired memory, skin aging and decreased muscle strength (12–14). It has been confirmed that CR can affect the expression level of 11β-HSD1 in fat, liver, muscle, and other organs of mice and pigs (15). Therefore, there may be a potential relationship between 11β-HSD1, CR and skeletal muscle aging. The study of 11β-HSD1 is helpful to clarify the mechanism of the anti-aging action of CR.

Thus, we used a CR model of elderly mice, combined with muscle-specific 11β-HSD1 knockout mice and 11β-HSD1 overexpression mice, to confirm that CR can delay muscle aging by inhibiting 11β-HSD1. Moreover, the proliferation and differentiation ability of MuSCs from these mice were observed *in vitro*. Together, we aimed to propose an effective regimen to improve the function of MuSCs that may be promising in sarcopenia protection during aging.

## 2. Materials and methods

### 2.1. Human samples

Circulating blood samples were taken from 440 people aged from 23 to 94 years. We collected blood samples from participants who had fasted overnight, centrifuged the samples at 4.0°C for 10 min at 1,000 rpm, and then analyzed the samples. Muscle biopsies from 20 adults aged 15 to 65 years were obtained from the vastus lateralis under general anesthesia during fracture surgery. The exclusion criteria were as follows: (1) Adults with musculoskeletal disorders, autoimmune disorders, thyroid dysfunction, other endocrine disorders (e.g., pituitary, adrenal, and parathyroid disorders), and other disorders; and (2) patients who took gonadal hormone, glucocorticoids, thyroid hormones, anti-seizure medication, anti-depressants or other drugs that affect muscle metabolism. All subjects and samples in this study were collected in accordance with the protocol approved by the Ethics Committee of the First Affiliated Hospital of Nanjing Medical University (No. 2019-SR-481). Informed consent of all subjects was obtained.

### 2.2. Muscle mass and function assessment

A dual-energy X-ray absorptiometry scanner (Hologic, Bedford, Massachusetts, USA) was used to measure total skeletal muscle mass as well as local muscle mass, such as that of the arms and legs. All scans were obtained by the same certified technician. The instrument used in this study has stable long-term performance (coefficient of variation <0.5%) and high-confidence *in vivo* precision.

The electronic grasping force instrument measures grip strength of the mice:mice were placed in the laboratory for 20 min to acclimate. Remove the mice from the cage and grasp the middle part of the mouse’s tail with the thumb and forefinger. After the mice forelimb grasps the sensing rod of the instrument, drag the mouse tail parallel to the rear until the forelimb is released. The instrument automatically records the maximum grip during the process. This process was repeated three times and the maximum grip value was recorded.

### 2.3. Animal model

#### 2.3.1. Calorie restricted (CR) mice

19-month-old C57BL/6J male mice (Model Animal Research Center of Nanjing University) were reared on a 12-hour light/dark cycle. They were housed individually in cages and fed *ad libitum* with standard laboratory chow for 4 days. Mean intakes were estimated as 4-day daily food intakes. Then, they were randomly divided into two groups: normal diet (ND) and calorie restricted (CR). For the next 12 weeks, ND mice were maintained on an *ad libitum* diet. Gradually increase the degree of CR, starting with a 10% restriction in the first week, increasing to 25% in the second week and 40% in the remaining 10 weeks. Both groups had unlimited access to water. Food intake was monitored daily, and the animals were weighed weekly.

#### 2.3.2. 11β-HSD1 knock-in mice

The strategy we chose recommended to select Transcript Hsd11b1-201 (NM_017080) for presentation. The Hsd11b1-201 gene had six exons, the ATG start codon is in exon 1, and the exon 6 contains the TAG stop codon. We generated H11-CAG-Hsd11b1-flag-PolyA knock-in mice by the CRISPR/Cas9 system. Co-inject Cas9 mRNA, sgRNA, and donor into fertilized eggs. The sgRNA directs Cas9 endonuclease to cut at the H11 locus and creates a DSB (double-strand break). This breaks were repaired and leads to the insertion of in CAG-Hsd11b1-flag-PolyA into the H11 locus.

#### 2.3.3. 11β-HSD1 knockout mice

The strategy we chose recommended to select Transcript Hsd11b1-001 for presentation. Approximately 0.7 kb of the genomic region was removed *via* Cre/LoxP excision. The absence of exon 3 caused a frame shift that ultimately disrupted the protein domain that followed. Approximately 29 N-terminal residues remained intact.

#### 2.3.4. MyoD-cre 11β-HSD1 knockout mice

11β-HSD1^flox/flox^ mice were crossed with MyoD-Cre mice obtained from Shanghai Institute of Biochemistry and Cell Biology. Offspring were intercrossed to generate MyoD-cre 11β-HSD1^–/–^ mice. 11β-HSD1^flox/flox^ littermates were used as controls.

All experimental protocols for animals in this study were reviewed and approved by the Animal Care Committee of the Model Animal Research Center of Nanjing University and in compliance with the Institutional Animal Care and Use Committee guidelines.

### 2.4. Muscle satellite cell isolation and differentiation

Mouse muscle stem cells (MuSCs) were isolated by a preliminary research protocol (16). Briefly, All hindlimb muscles of mice were dissected and scratched to pieces. Muscles were digested in wash medium (Ham’s F10 with 10% horse serum) containing collagenase II (800 units/ml) for 60 min at 37°C. Digested muscles were washed in washing medium, then collagenase II (80 units/ml) and dispase (1 unit/ml) were added for further digestion for 30 min. The resulting suspensions were pipetted 15 times with a 20G needle of a syringe and then filtered with a 40-μm cell strainer. FACS analysis was performed, and cells with Sca1-/CD11-/CD31-/CD45-/VCAM1 + signals represent the population of MuSCs. All antibodies used in FACS analysis were used at a dilution of 1:75. MuSC sorting was performed by using the BD Influx cell sorter (BD Biosciences). MuSCs were cultured on collagen-coated dishes in Ham’s F-10 Nutrient Mixture (Gibco) [20% FBS, 2.5 ng/ml bFGF (Invitrogen)] and T-cell conditioned medium (F10 medium with 20% FBS:T-cell medium = 50:50). Cultures were passaged when they reached 60–70% confluence.

The medium was replaced to differentiation medium (DMEM) containing 2% horse serum to induce cells differentiation. Cells could be maintained in differentiation medium for 72 h. BVT.2733 is a 11β-HSD1 selective inhibitor synthesized by China Pharmaceutical University based on patent information.

### 2.5. MTT assay

The Cell vaccination in 96-well plates (with equal number of cells in each well), and the cell viability was measured by MTT assay daily for consecutive 5 days. Briefly, add 20 μl MTT working solution (5 mg/ml) to each well, and incubate the plates at 37°C for 4 h. Then, remove the supernatants, and the resultant MTT formazan was dissolved in 100 μl of DMSO. Absorbance measurements at 595 and 630 nm wavelengths.

### 2.6. Myofiber diameter and cross-sectional area measurement

Using Adobe Acrobat 9 pro software (Adobe) to measure the myofiber diameter. Three independent visual fields were randomly selected in each sample. Each treatment measured 300 fibers from each field of view. Measure the myofiber cross-sectional area by ImageJ software (NIH). Three independent visual fields in each sample were chosen randomly. Three hundred myofibers from each visual field were measured for analysis. The person who performed the measurement was blinded to the identity of the sample.

### 2.7. Gene expression analysis

Total RNA was isolated using RNeasy kits (Qiagen). 1 μg of total RNA per sample was reverse transcribed to cDNA by MuLV transcriptase (NEB) and oligo dT primers following the manufacturer’s instructions. Briefly, RNA was denatured at 85°C for 3 min. MuLV reverse transcriptase was subsequently added to the mixture and incubation under 42°C for 1 h. Three replicate quantitative PCR (qPCR) reactions were performed in a Bio-Rad thermocycler system (Bio-Rad) by using SYBR Green PCR master mix (DBI). Data were analyzed with iQ5 optical system software (Bio-Rad).

### 2.8. Western blot method

Four mice were decapitated with 1% sodium pentobarbital and muscle was immediately collected. Tissues and cells were homogenized in a lysis buffer containing a protease inhibitor mixture, centrifuged at 12,000 × *g* for 15 min, and supernatant was collected. The proteins were isolated using SDS-PAGE gel (10% separation gel) and transferred to NC membranes. The membranes were blocked at room temperature with 5% degreased emulsion for 1 h and incubated with the respective master antibody at 4°C overnight:11β-HSD1 (ab169785, 1:1000, Abcam, Cambridge, United Kingdom), MuRF1 [MuRF1 (C-11): sc-398608, 1:200; SANTA CRUZ BIOTECHNOLOGY, INC.], Atrogin1 [MAFbx (F-9): SC-166806, 1:200; SANTA CRUZ BIOTECHNOLOGY, INC.], and GAPDH (MC4, #RM2002, 1:10000; Ray Antibody Biotech). After three washes with TBST, Horseradish peroxidase (HRP) is combined with goat anti-rabbit Antibody (#RM3002, 1:10000, Ray Antibody Biotech) or goat anti-mouse antibody (#RM3001, 1:10000, Ray Antibody Biotech). The protein was detected by ECL (Bio-Rad) method.

### 2.9. Immunofluorescent staining

Cells or sections were fixed with 4% paraformaldehyde and then permeabilized with cold methanol, along with anti-MYHC (Merck Millipore, clone 05-716, 1:1000) and anti-lamnin (Abcam, clone B00648, 1:1000) antibodies as primary antibodies. Cells and sections were subsequently stained with Alexa 488-, 561- or 647-labeled anti-mouse or -rabbit antibodies (Invitrogen). All images were obtained by confocal microscopy (Leica).

### 2.10. Measurement of oxygen consumption

Differentiated cell’s oxygen consumption rates (OCRs) were measured using an XF24 respirometer (Seahorse Bioscience, Santa Clara, California, USA). Basal oxygen consumption as well as oxygen consumption in the presence of drugs that disrupt the mitochondrial respiratory chain were measured: oligomycin (OL, ATP synthase inhibitor, 1 mM) and carbonyl cyanide-4-(trifluoromethoxy) phenylhydrazone (FCCP, uncoupler, 1 mM). Finally, mitochondrial respiration was blocked with rotenone (Rot, 1 mM) (Sigma–Aldrich).

### 2.11. Statistical analysis

All data are expressed as the mean ± SEM. Comparisons between groups were analyzed using an unpaired Student’s *t*-test by GraphPad Prism. One-way ANOVA with Tukey *post-hoc* analysis was used for comparisons between multiple groups. The results of the analysis and statistical significance are presented in the Figures and Figure Legends. A *P*-value < 0.05 was considered significant. Every experiment was repeated independently.

## 3. Results

### 3.1. The 11β-HSD1 expression level is up-regulated in human muscles with age-related atrophy

**FIGURE 1:**
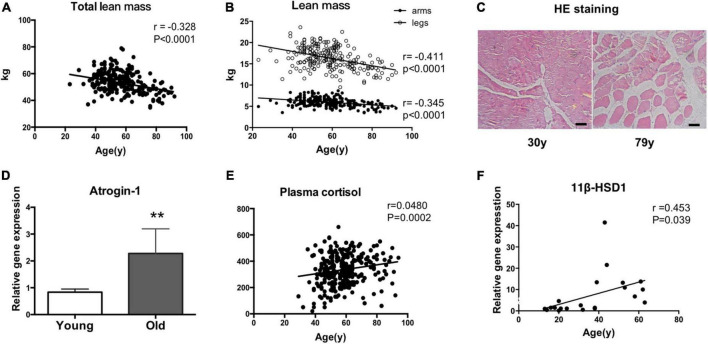
11β-HSD1 expression is up-regulated in human muscles with age-related atrophy. **(A)** Total and regional lean mass (arms and legs) were obtained from the DXA measurement. Total lean mass was negatively correlated with age (*r* = –0.328, *P* < 0.0001). **(B)** With aging, the muscles of the lower limbs decline faster than those of the upper limbs (legs: *r* = –0.411, *P* < 0.0001; arms: *r* = –0.345, *P* < 0.0001). **(C)** Representative HE staining images of muscle fibers in a 79-year-old man and a 30-year-old man. Scale bars, 50 μm. **(D)** RT–PCR assays were performed with vastus lateralis muscles obtained from young (aged < 30 years, *n* = 7) or old people (aged > 60 years, *n* = 8), and the results were normalized to GAPDH. **p < 0.01. **(E)** Plasma cortisol levels were examined by ELISA in blood samples taken from recruited people (*n* = 440). **(F)** The mRNA level of 11β-HSD1 in vastus lateralis muscles was positively correlated with age (*r* = 0453, *p* = 0.039) (*n* = 20).

Total and regional lean mass (arms and legs) were obtained from the DXA measurement. Pearson correlation analysis showed that total lean mass was negatively correlated with age (*r* = −0.328, *P* < 0.0001) (Figure 1A). In addition, as shown in Figure 1B, the decline rate of sarcopenia caused by aging in all regions of the body was not consistent. With aging, the muscles of the lower limbs declined faster than those of the upper limbs (legs: *r* = −0.411, *P* < 0.0001; arms: *r* = −0.345, *P* < 0.0001) (Figure 1B). The muscle fiber size was also examined. Muscle fiber size seemly was reduced in a 79-year-old man compared with a 30-year-old man, suggesting the possible occurrence of muscle atrophy (Figure 1C). Consistently, the expression levels of atrogin-1 were increased in the vastus lateralis muscles of older people compared with young people, as indicated by RT–qPCR (Figure 1D). Numerous studies have shown that long-term stress accelerates aging. Cortisol is a stress hormone, and the plasma cortisol level increased with aging (Figure 1E). As an enzyme that locally activates glucocorticoids, the mRNA level of 11β-HSD1 in vastus lateralis muscles was positively correlated with age (*r* = 0453, *p* = 0.039) (Figure 1F).

### 3.2. CR may improve muscle atrophy and muscle function in aged mice by reducing 11β-HSD1

**FIGURE 2:**
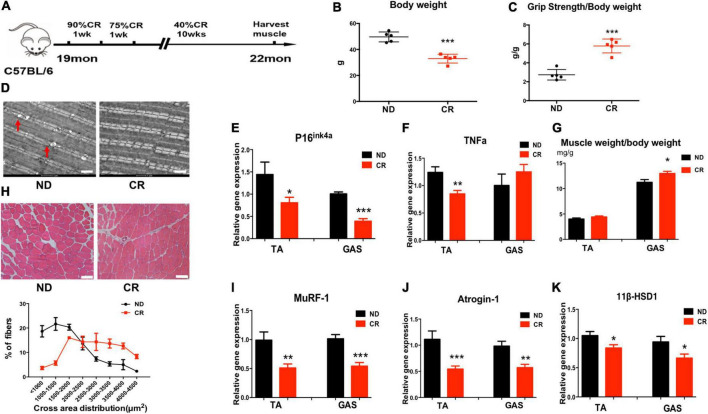
Calorie restriction (CR) improves muscle atrophy and muscle function in aged mice by reducing 11β-HSD1. **(A)** CR was initiated in mice at 19 months of age and continued for 3 months to 22 months of age. **(B)** Body weight of CR mice and normal diet (ND) mice (*n* = 5 mice per group). **(C)** Muscle strength of CR mice and ND mice (*n* = 5 mice per group). **(D)** Representative images using electron microscopy for vastus lateralis muscles from ND and CR mice. Scale bars, 2 μm. **(E,F)** mRNA quantification of the senescence marker gene p16^ink4a^ and the inflammation gene TNFα. **(G)** Muscle weight/body weight in CR and ND mice (*n* = 5 mice per group). **(H)** Representative HE staining images of muscle cross sections derived from CR and ND mice. Scale bars, 50 μm. Percentage distribution of muscle fiber cross-sectional area derived from muscles (*n* = 5 mice per group). **(I,J)** mRNA levels of the muscle atrophy factors atrogin-1 and MuRF-1 in both TA and GAS (*n* = 5 mice per group). **(K)** mRNA quantification of the 11β-HSD1 genes in both TA and GAS (*n* = 5 mice per group). *p < 0.05, **p < 0.01, ***p < 0.001.

To explore the function of CR in sarcopenia, we analyzed mice under CR initiated at 19 months of age and continued for 3 months to 22 months of age (Figure 2A). Then, we found that body weight was decreased in CR mice compared to normal diet (ND) mice (Figure 2B). Furthermore, we studied muscle strength and function in both the ND and CR groups of mice. We noticed that grip strength/body weight increased in CR mice compared with ND mice (Figure 2C). High-energy muscle contains a large number of mitochondria. Fat deposition will mostly produce toxic lipid intermediates through anaerobic metabolic pathways, which will eventually lead to mitochondrial dysfunction, increased endoplasmic reticulum pressure, and even cell death. Using electron microscopy, we found that CR could reduce lipid droplets in muscle; however, an increased number of droplets could be found in ND aged mice, suggesting that CR reduced mitochondrial damage (Figure 2D). Based on previous observations, the mRNA expression levels of the senescence marker gene p16^ink4a^ and the inflammation gene TNFα were low in aged CR mice (Figures 2E, F).

In response to CR, we noticed that muscle weight/body weight increased in CR mice compared with ND mice (Figure 2G). The size of the muscle fiber in CR mice was larger than that in ND mice (Figure 2H). Moreover, the mRNA levels of the muscle atrophy factors atrogin-1 and MuRF-1 were down-regulated in both TA and GAS (Figures 2I, J). To determine whether CR plays a role in delaying muscle atrophy through 11β-HSD1, we examined 11β-HSD1 expression levels in muscles. The expression level of 11β-HSD1 was markedly decreased in both the TA and GAS of CR mice compared with ND mice (Figure 2K). The western blot results of Atrogin1, MuRF1, and 11β-HSD1 were included in Supplementary Figure 1. These results revealed the correlation between the down-regulation of 11β-HSD1 in muscle and CR, suggesting that 11β-HSD1 may be the target of CR.

### 3.3. Overexpression of 11β-HSD1 leads to muscle atrophy in young mice

**FIGURE 3:**
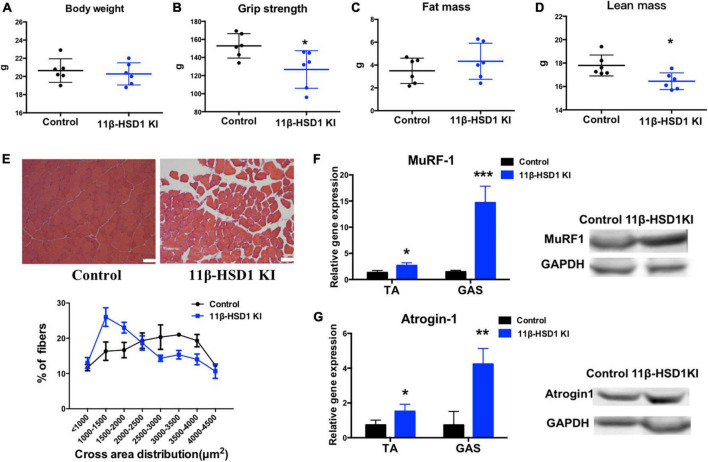
Overexpression of 11β-HSD1 leads to muscle atrophy in young mice. **(A)** Body weight of 11β-HSD1 KI mice and control mice (*n* = 6 mice per group) at 2 months of age. **(B)** Muscle strength of 11β-HSD1 KI mice and control mice (*n* = 6 mice per group). **(C)** Fat mass of 11β-HSD1 KI mice and control mice (*n* = 6 mice per group). **(D)** Lean mass of 11β-HSD1 KI mice and control mice (*n* = 6 mice per group). **(E)** Representative HE staining images of muscle cross sections derived from 11β-HSD1 KI mice and control mice. Scale bars, 50 μm. Percentage distribution of muscle fiber cross-sectional area derived from muscles. **(F,G)** mRNA and protein levels of the muscle atrophy factors atrogin-1 and MuRF-1 in both TA and GAS (*n* = 6 mice per group). TA, Tibialis anterior muscle; GAS, gastrocnemius. *p < 0.05, **p < 0.01, ***p < 0.001.

Considering that 11β-HSD1 may cause muscle atrophy, we constructed an 11β-HSD1-overexpressing mouse model. The identification of overexpressed mice is included in the Supplementary Figure 2. No differences of body weight, fat mass and lean mass were observed between 11β-HSD1-overexpressing mice and control mice (Figures 3A, C, D); however, grip strength decreased in 11β-HSD1-overexpressing mice compared with control mice (Figure 3B). To further explore whether the elevated 11β-HSD1 level could induce muscle atrophy *in vivo*, we examined lean mass and fiber size and found decreased lean mass and smaller muscle fiber size in 11β-HSD1-overexpressing mice (Figure 3E). Consistent with the morphological changes, the expression levels of atrogin-1 and MuRF1 were significantly increased in overexpression mice, as indicated by RT–qPCR and western blot (Figures 3F, G). These results suggested that 11β-HSD1 can induce atrophy in young mice.

### 3.4. Muscle-specific 11β-HSD1 knockout rescued age-related muscle atrophy in aged mice

**FIGURE 4:**
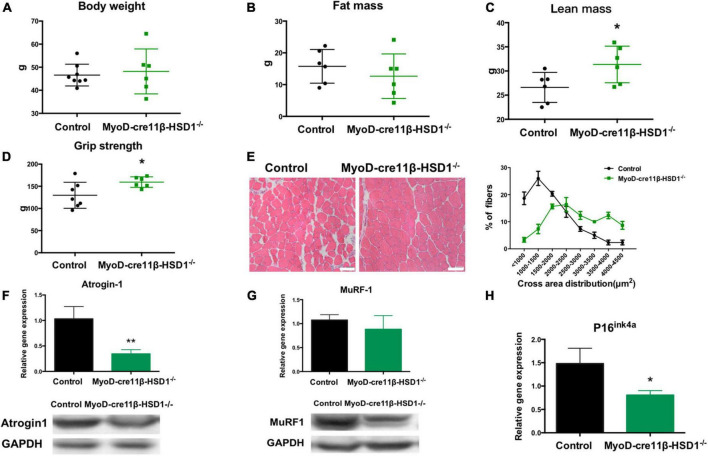
Muscle-specific 11β-HSD1 knockout rescued age-related muscle atrophy in aged mice. **(A)** Body weight of MyoD-cre 11β-HSD1 KO mice (*n* = 6) and control mice (*n* = 8) at 22 months of age. **(B)** Muscle strength of MyoD-cre 11β-HSD1 KO mice (*n* = 6) and control mice (*n* = 6). **(C)** Lean mass of MyoD-cre 11β-HSD1 KO mice (*n* = 6) and control mice (*n* = 6). **(D)** Fat mass of MyoD-cre 11β-HSD1 KO mice (*n* = 6) and control mice (*n* = 7). **(E)** Representative HE staining images of muscle cross sections derived from muscle-specific 11β-HSD1 knockout mice and control mice. Scale bars, 50 μm. Percentage distribution of muscle fiber cross-sectional area derived from muscles. **(F,G)** mRNA and protein levels of the muscle atrophy factors atrogin-1 and MuRF-1 in muscles (*n* = 6 mice per group). **(H)** mRNA quantification of the senescence marker gene p16^ink4a^ in muscles (*n* = 6 mice per group). *p < 0.05, **p < 0.01.

To investigate whether reducing the 11β-HSD1 expression level could rescue muscle atrophy in old mice, we generated muscle-specific 11β-HSD1 knockout mice (MyoD-Cre11β-HSD1^–/–^) by crossing MyoD-cre mice with 11β-HSD1 flox/flox mice (Supplementary Figure 2). 11β-HSD1^flox/flox^ littermates were used as controls. Mice at 22 months of age were healthy. No tumors, obesity, injury, or other diseases were detected. Although the body weight and fat mass of the mice were not different between the control and MyoD-cre 11β-HSD1^–/–^ mice (Figures 4A, B), both the lean mass and muscle strength were increased in muscle-specific 11β-HSD1 knockout mice (Figures 4C, D). Figure 4E shows representative HE staining images of muscle cross sections, and muscle-specific 11β-HSD1 knockout delayed muscle atrophy in 20-month-old mice (Figure 4E). We further found that the gene and protein expression levels of the muscle atrophy factors atrogin-1 and MuRF1 were down-regulated upon muscle-specific 11β-HSD1 knockout (Figures 4F, G). Additionally, the mRNA level of the senescence marker gene p16^ink4a^ in muscles was decreased (Figure 4H). Considering that MyoD is one of the main markers of muscle stem cells (MuSCs), these data demonstrated that 11β-HSD1 may rescue age-related muscle atrophy by maintaining the function of MuSCs.

### 3.5. The differentiation ability of MuSCs in 11β-HSD1 KO mice and CR mice was improved

**FIGURE 5:**
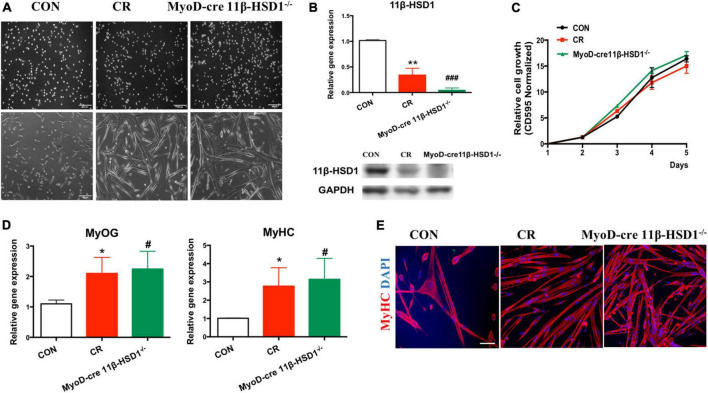
The differentiation ability of mouse muscle stem cells (MuSCs) in MyoD-cre 11β-HSD1 KO and CR mice was improved. **(A)** MuSCs were isolated from CR and MyoD-cre 11β-HSD1^–/–^ and control mice at 22 months of age. Muscle-specific 11β-HSD1 knockout mice and CR mice displayed better differentiation ability, as indicated by their larger myofibers. Scale bars, 200 μm. **(B)** Gene and protein expression levels of 11β-HSD1 in MuSCs of CR and MyoD-cre 11β-HSD1^–/–^ and control mice. **(C)** The proliferation rates of MuSCs sorted from 22-month-old CR and 11β-HSD1^–/–^ mice and their wild-type counterparts were detected by MTT assay. **(D)** Gene expression levels of MyHC and MyOG in 11β-HSD1^–/–^ mice and CR mice during the differentiation of MuSCs. *p < 0.05, **p < 0.01; #*p* < 0.01, ###*p* < 0.001. *CR mice compared with CON; #MyoD-cre 11β-HSD1^–/–^ mice compared with CON. **(E)** Representative immunofluorescent staining images of cultured MuSCs at 3 days of differentiation from CR and MyoD-cre 11β-HSD1^–/–^ and control mice at 22 months of age. Red indicates MyHC staining; DAPI indicates nuclei. Scale bars, 200 μm.

To further confirm that 11β-HSD1 plays essential roles in maintaining the function and homeostasis of MuSCs, we first isolated MuSCs from CR and 11β-HSD1^–/–^ mice and checked the effects of 11β-HSD1 on MuSCs (Figure 5A). As expected, 11β-HSD1 expression was significantly decreased in the MuSCs of both 11β-HSD1 knockout and CR mice (Figure 5B). Notably, there was no obvious difference in the proliferation rates of MuSCs sorted from 22-month-old CR and 11β-HSD1^–/–^ mice and their wild-type counterparts detected by MTT assay (Figure 5C). However, we observed that muscle-specific 11β-HSD1 knockout mice and CR mice displayed better differentiation ability, as indicated by larger myofibers (Figure 5A). In addition, both 11β-HSD1 knockout mice and CR mice showed increased expression levels of MyHC and MyOG during the differentiation of MuSCs (Figure 5D). Consistent with the results of gene, both 11β-HSD1 knockout and CR mice showed increased immunofluorescence staining of MyHC (Figure 5E). Taken together, these data demonstrated that inhibition of 11β-HSD1 expression promoted myoblast differentiation. CR may promote MuSC differentiation and delay muscle atrophy by down-regulating 11β-HSD1 in MuSCs.

### 3.6. 11β- HSD1 inhibitor BVT.2733 improved mitochondrial function in MuSCs

**FIGURE 6:**
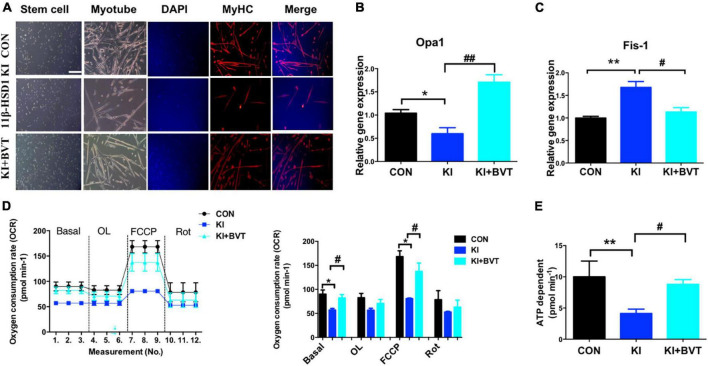
11β-HSD1 inhibitor BVT.2733 improved mitochondrial function in mouse muscle stem cells (MuSCs). **(A)** Representative immunofluorescent staining images of cultured MuSCs at 3 days of differentiation from 11β-HSD1 KI mice and 11β-HSD1 inhibitor BVT.2733-treated KI mice and control mice at 2 months of age. Red indicates MyHC staining; DAPI indicates nuclei. Scale bars, 200 μm. **(B)** mRNA levels of the mitochondrial fusion gene Opa1 in MuSCs from 11β-HSD1 KI mice and 11β-HSD1 inhibitor BVT.2733-treated KI mice and control mice. **(C)** mRNA levels of the mitochondrial cleavage gene Fis-1 in MuSCs. **(D)** Oxygen consumption rate measurement during the differentiation of MuSCs from 2-month-old mice. **(E)** ATP synthesis rate measurement during the differentiation of MuSCs from 2-month-old mice. *p < 0.05, **p < 0.01; #*p* < 0.01, ##*p* < 0.01. *KI mice compared with control; #KI + BVT compared with control.

Mitochondria are an important production unit of skeletal muscle and an important production site of oxidative phosphorylation and ATP synthesis, known as the “power house” (17). Consistent with our previous results, 11β-HSD1-overexpressing mice displayed impaired myoblast fusion and myotube formation in MuSCs. As shown in Figure 6A, we labeled MyHC with fluorescent staining to detect myogenic conditions and found 11β-HSD1-overexpressing mice displayed impaired myoblast fusion and myotube formation in MuSCs, suggesting that the myogenic process was inhibited by 11β-HSD1 during differentiation. After treatment with the 11β-HSD1 inhibitor BVT.2733, myotube formation increased. Furthermore, the MuSCs were collected, and the levels of mitochondrial fusion and cleavage genes were detected by RT–PCR. We observed that the fusion gene Opa1 was decreased and the cleavage gene Fis-1 was increased in 11β-HSD1-overexpressing mice, and treatment with the inhibitor BVT.2733 reversed this change (Figures 6B, C). Importantly, overexpression of 11β-HSD1 led to a marked decrease in mitochondrial oxygen consumption under basal conditions and after stimulation with FCCP, as measured by OCR (Figure 6D), which was consistent with the reduction in mitochondrial ATP compatibility levels. Additionally, the 11β-HSD1 inhibitor BVT.2733 increased ATP synthesis in MuSCs (Figure 6E). Collectively, these results suggest that inhibition of 11β-HSD1 in MuSCs may improve mitochondrial function.

## 4. Discussion

**FIGURE 7:**
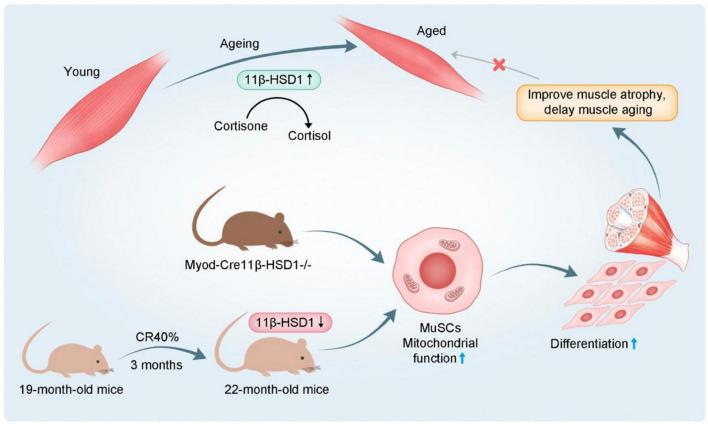
Schematic diagram 1.11β-HSD1 expression level was increased in age-related muscle atrophy. 2. Muscle-specific 11β-HSD1 knockout rescued age-related muscle atrophy. 3. CR promoted the mitochondrial function and differentiation ability of MuSCs by reducing 11β-HSD1, thereby delaying muscle atrophy in aged mice.

In the present study, we showed that 11β-HSD1 expression levels were increased in age-related muscle atrophy. CR may improve muscle atrophy and muscle function in aged mice by reducing 11β-HSD1 (Figure 7). So far, CR is the only known method considered to extend lifespan and reduce the age-related diseases (18). A study in 2010 at the University of Wisconsin Primate Center showed that CR significantly reduced age-related mortality in monkeys compared with control animals (19). In addition, the CR group also had significantly lower rates of sarcopenia, type 2 diabetes, cancer, and cardiovascular disease (20). Currently, there are few studies on CR in sarcopenia and they have conflicting results (21). Although most studies suggested that CR is beneficial to muscle metabolism, it has also been reported that CR may promote age-related muscle loss, lower body mass index, and increase the risk of disability and mortality in the elderly (18). It is worth noting that different levels of nutrition, age at initiation of CR, duration of caloric restriction and changes in dietary macronutrient content are all potentially important factors in CR (22, 23). Thus, we speculated that CR improves sarcopenia in elderly individuals with good nutritional status. In our study, 40% CR did not cause malnutrition in older mice. Additionally, we compared our CR diet with the micronutrient standards set by the National Research Council for Laboratory Mice and found that our young CR mice continued to meet the micronutrient standards. Therefore, our findings demonstrated promising sarcopenia protection with 40% CR in older mice.

The mechanism of CR delaying sarcopenia is unclear. Current studies generally study the regulation of mitochondrial function, inflammation, oxidative stress, apoptosis and autophagy (24). Glucocorticoid (GC) is a stress hormone, and studies have shown that 24-hour urine GC output in elderly individuals is related to DNA damage (25). A follow-up study of nearly 3,000 adults conducted by Dutch researchers showed that GC levels are related to shortening of telomere length in peripheral white blood cells, which can aggravate aging (26). Some studies have found that GCs can directly inhibit the function of muscle stem cells and lead to muscular atrophy by up-regulating myostatin (27). Thus, the reduction in GC secretion may be a way to improve sarcopenia. As the local amplifier of GC, 11β-HSD1 is expressed in muscle, fat, heart, brain, skin, and other tissues (11). Some studies have found that brain 11β-HSD1 can increase the local GC level in brain tissue, thereby promoting brain aging and damaging memory function in mice (12). The expression level and activity of the 11β-HSD1 gene are also significantly increased in elderly skin, promoting skin aging (13). Increased expression of 11β-HSD1 in muscle tissue of elderly individuals is significantly correlated with decreased muscle strength (14). In our study, we first found that 11β-HSD1 plays a key role in the relationship between CR and sarcopenia and further found that muscle-specific knockout of 11β-HSD1 could delay muscle atrophy and improve muscle function in aged mice. Thus, confirmation of 11β-HSD1 as a key regulator of aging-related muscle atrophy has important therapeutic significance for sarcopenia.

Another novel finding was that CR can promote the differentiation of MuSCs by down-regulating 11β-HSD1. As the largest organ in the body, skeletal muscle accounts for approximately 40–50% of the adult male’s total weight (28). Sarcopenia caused by aging is a multilayered and complex process involving many factors (29, 30). Changing one factor alone cannot prevent or cure sarcopenia. MuSCs are responsible for skeletal muscle regeneration. When the muscle is damaged, resting MuSCs are activated and differentiate and fuse to form muscle tubes, which are arranged in order and fuse to form muscle fibers. Currently, MuSCs are the most ideal seed cells for cell therapy (2). The fusion of MuSCs is the most important contributor to and participant in muscle regeneration (31). Here, we used 11β-HSD1-overexpressing and knockout mice and CR mice for primary MuSC culture. The differentiation ability of MuSCs in 11β-HSD1 KO mice and CR mice was significantly improved. In contrast, the differentiation ability in overexpression mice was significantly reduced. CR can improve the function of MuSCs and delay muscle aging, which is an important direction for the prevention of sarcopenia in elderly individuals.

The life activities of cells depend on mitochondria to provide energy and participate in various activities in the body (32). A previous study found that mitochondria play an important role in maintaining the pluripotency of stem cells and inducing differentiation, and with differentiation, mitochondrial aerobic metabolism gradually becomes dominant (33). In recent years, mitochondrial dysfunction has been proposed as a biomarker of muscle stem cell senescence (34). The cell energy metabolizer is programmed to control the addition of drugs to the culture medium at a specific time to measure the increase in the rate of oxygen consumption under different conditions. FCCP is the uncoupling agent of mitochondrial oxidized phosphoric acid that causes the mitochondrial membrane to depolarize and form proton leakage, increasing the oxygen consumption of mitochondria to reach the maximum respiration rate (35). Here, we assessed mitochondrial function by measuring mitochondrial oxygen consumption and calculated mitochondrial ATP production capacity. Overexpression of 11β-HSD1 led to a marked decrease in mitochondrial oxygen consumption under basal conditions and after stimulation with FCCP, which was consistent with the reduction in mitochondrial ATP compatibility levels. Additionally, mitochondrial function is closely related to structure. When mitochondrial fusion lysis activity in the cell is impaired, it leads to cellular functional defects (36, 37). We observed that the fusion gene Opa1 was decreased and the cleavage gene Fis-1 was increased in 11β-HSD1-overexpressing mice, and treatment with the inhibitor BVT.2733 reversed this change. It will be interesting to determine the specific mechanisms by which 11β-HSD1 regulates mitochondrial function in skeletal muscle during aging in the future.

There were certain limitations to this study. For example, since nutrition and physical activity are two important factors affecting sarcopenia, we believe that being able to add physical activity of mice is more complete for the whole study. Secondly, western blot or fluorescent immunohistochemistry data should be added for Opa1 and Fis-1 and Inflammatory factors TNFα and aging gene p16. Thirdly, considering the level of GC is closely associated with 11β-HSD1, we should not only focus on the expression level of 11β-HSD, but also the expression level of GC. Moreover, muscle weight, body weight and muscle strength have complicated relationships, and it is meaningful to study the relationship between them. Also, in order to avoid selection bias, we should try to expand the sample size especially MyoD-Cre 11β-HSD1 knockout mice. These shortcomings merit further study.

In conclusion, our findings highlight that increased 11β-HSD1 is a hallmark of aged muscles. Furthermore, CR in aged mice reduced the local effective concentration of GC through 11β-HSD1, thereby promoting mitochondrial function and the differentiation ability of MuSCs (Figure 7). Together, we speculate that targeting an 11β-HSD1-dependent metabolic pathway may represent a novel strategy for developing therapeutics against age-related muscle atrophy.

## Data availability statement

The raw data supporting the conclusions of this article will be made available by the authors, without undue reservation.

## Ethics statement

The studies involving human participants were reviewed and approved by the Ethics Committee of the First Affiliated Hospital of Nanjing Medical University (protocol code 2019-SR-481 and 2020-01-07). Written informed consent to participate in this study was provided by the participants or their legal guardian/next of kin.

## Author contributions

SL designed the experiments, conducted the study, and wrote the manuscript. QS performed data analysis and mapping. HL and WX performed animal model and experimental operation. QC obtained tissue specimens. YL, XW, and GD performed project administration and supervision. All authors have read and agreed to the published version of the manuscript.
