# Supplementary material for: Caloric restriction delays age-related muscle atrophy by inhibiting 11β−HSD1 to promote the differentiation of muscle stem cells
Source: Front Med (Lausanne). 2023 Jan 5;9:1027055. doi: 10.3389/fmed.2022.1027055 (PMC9849809; doi:10.3389/fmed.2022.1027055)
Supplement: Supplementary file 1 [file Image_1.PDF]

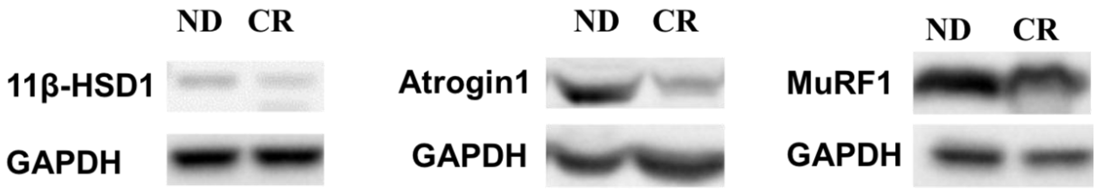

Figure S1. protein expression levels of 11β-HSD1 in ND mice and CR mice

ND： normal diet

CR： Calorie restriction

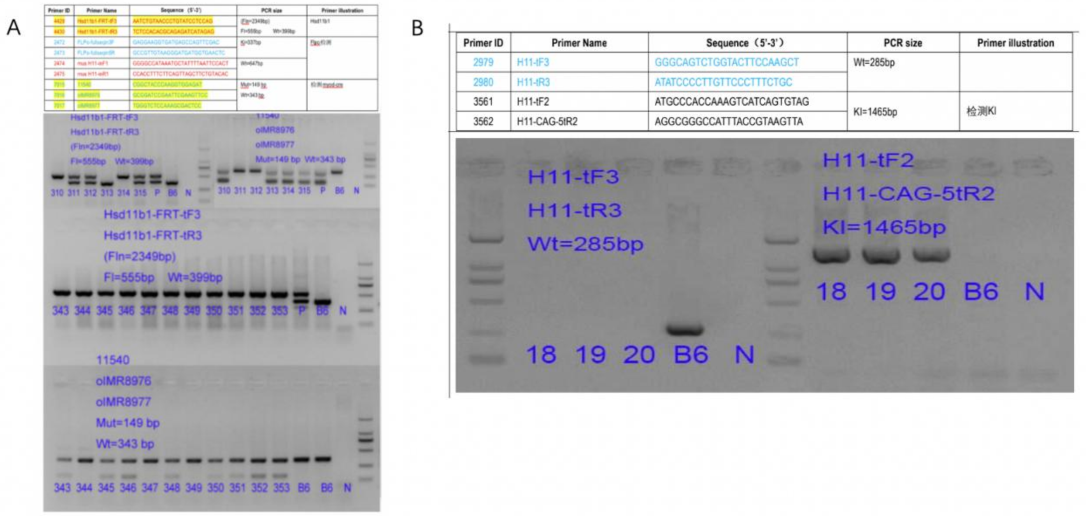

Figure S2. （A）agarose gel identification for genotyping data of muscle-specific 11β-HSD1 KO mice （B） garose gel identification for genotyping data of 11β-HSD1 KI mice

11β-HSD1fl/fl mice (genotype 11β-HSD1:fl/fl, Cre:wt/wt): 329, 333, 344, 347, 349. Muscle-specific 11β-HSD1 KO mice (genotype 11β-HSD1: fl/fl, Cre: mut/wt): 310, 314, 343, 345, 346, 348, 350, 351, 352, 353.

P: positive control

N: negative control
